# Nursing and midwifery regulatory reform in east, central, and southern Africa: a survey of key stakeholders

**DOI:** 10.1186/1478-4491-11-29

**Published:** 2013-06-25

**Authors:** Carey F McCarthy, Joachim Voss, Marla E Salmon, Jessica M Gross, Maureen A Kelley, Patricia L Riley

**Affiliations:** 1 Center for Global Health, Division of Global HIV/AIDS, U.S. Centers for Disease Control and Prevention (CDC), 1600 Clifton Road, MS-E30, Atlanta, GA, 30333, USA; 2 Department of Global Health, University of Washington School of Nursing, 1959 NE Pacific Avenue, Seattle, WA, 98195, USA; 3 University of Washington Evans School of Public Affairs, 4100 15th Ave NE, Seattle, WA, 98195, USA; 4 ARC Project Coordinator, Nairobi, Kenya; 5 Emory University Nell Hodgson Woodruff School of Nursing, 1520 Clifton Road, Atlanta, GA, 30322, USA

**Keywords:** Sub-Saharan Africa, Education, HIV, Midwifery, Nursing, Regulation, Stakeholders, Task sharing, Task shifting

## Abstract

**Background:**

In sub-Saharan Africa, nurses and midwives provide expanded HIV services previously seen as the sole purview of physicians. Delegation of these functions often occurs informally by shifting or sharing of tasks and responsibilities. Normalizing these arrangements through regulatory and educational reform is crucial for the attainment of global health goals and the protection of practitioners and those whom they serve. Enacting appropriate changes in both regulation and education requires engagement of national regulatory bodies, but also key stakeholders such as government chief nursing officers (CNO), professional associations, and educators. The purpose of this research is to describe the perspectives and engagement of these stakeholders in advancing critical regulatory and educational reform in east, central, and southern Africa (ECSA).

**Methods:**

We surveyed individuals from these three stakeholder groups with regard to task shifting and the challenges related to practice and education regulation reform. The survey used a convenience sample of nursing and midwifery leaders from countries in ECSA who convened on 28 February 2011, for a meeting of the African Health Profession Regulatory Collaborative.

**Results:**

A total of 32 stakeholders from 13 ECSA countries participated in the survey. The majority (72%) reported task shifting is practiced in their countries; however only 57% reported their national regulations had been revised to incorporate additional professional roles and responsibilities. Stakeholders also reported different roles and levels of involvement with regard to nursing and midwifery regulation. The most frequently cited challenge impacting nursing and midwifery regulatory reform was the absence of capacity and resources needed to implement change.

**Discussion:**

While guidelines on task shifting and recommendations on transforming health professional education exist, this study provides new evidence that countries in the ECSA region face obstacles to adapting their practice and education regulations accordingly. Stakeholders such as CNOs, nursing associations, and academicians have varied and complementary roles with regard to reforming professional practice and education regulation.

**Conclusion:**

This study provides information for effectively engaging leaders in regulatory reform by clarifying their roles, responsibilities, and activities regarding regulation overall as well as their specific perspectives on task shifting and pre-service reform.

## Background

In sub-Saharan Africa, the greater number and availability of nurses relative to physicians has resulted in health services delivery strategies which rely on nurses and midwives to perform tasks previously performed by physicians [1-4]. The widespread utilization of nurses and midwives functioning in expanded roles is seen as central to scaling-up HIV services in sub-Saharan Africa and strengthening related health systems [5-7]. However, the lack of supportive regulation, education, and legislation normalizing the expanded roles of nurses and midwives is a detriment to the sustainability of their contributions within their countries and across Africa [7-10]. Health professional regulation establishes the scope and standards for health worker practice and education, thereby ensuring their competency to provide health services [11-13]. In many countries in Africa, health professional regulation has not kept up with advancements in practice and education brought about by task shifting and pre-service reform [8,14-16]. For example, nursing and midwifery scopes of practice and pre-service curricula may not reflect new responsibilities associated with diagnosing HIV infections and prescribing antiretroviral treatment (ART) - tasks previously seen as the sole purview of physicians [17-19]. This situation has prompted a greater focus on nursing and midwifery regulatory bodies, or councils, to review and revise key practice and education regulations [20-22].

While many countries in this region have a nursing and midwifery council which is responsible for developing and implementing a regulatory framework, these groups do not always have sufficient capacity or resources to undertake regulation strengthening and reform [21-23]. Other nursing and midwifery policy, advocacy, and education stakeholders^a^ play an important role in adapting and institutionalizing these regulations [9,10,20]. Representatives of the ministry of health, professional associations, and nursing and midwifery academia are widely recognized as critical to introducing and sustaining professional reforms, such as task shifting (now referred to as task sharing, which emphasizes a team-lead and shared approach to client management) [7,22,24]. The chief nursing officer (CNO) in the ministry of health (MoH) is often responsible for national nursing workforce planning, developing/advising and enacting nursing policies, and providing nursing and midwifery services; the professional associations or unions represent the interests of practicing nurses and midwives; the academicians are responsible for the content and delivery of nursing and midwifery education. These stakeholder groups are critical facilitators to regulatory advancements, but can oppose changes or reform if not appropriately engaged in the process [25,26]. Unfortunately, there is little reported evidence documenting best practices for the engagement of partners and stakeholders in these processes. Given both the importance and urgency of regulatory reform, gaining better understanding of the activities, involvement and perspectives of those shaping regulation and practice is crucial to future progress in this area.

The US Centers for Disease Control and Prevention (CDC) conducted a survey of the CNO, the president of the nursing and midwifery professional association, and a representative of nursing and midwifery academia from 13 countries in east, central, and southern Africa (ECSA). The purpose of the survey was to elicit stakeholder perceptions of their roles, engagement, and, most importantly, their perspectives on adapting regulations governing nursing and midwifery practice and education. Although this information is fundamental to understanding how best to strategically involve stakeholders in advancing and strengthening nursing and midwifery practice, querying these leaders had not occurred previously on a regional basis.

## Methods

The investigators invited CNOs, presidents of national nursing and midwifery associations, and nursing and midwifery educational leaders from 13 countries in the ECSA region to participate in a written survey presented at a meeting of the African Health Profession Regulatory Collaborative for Nurses and Midwives (ARC) in Nairobi, Kenya, on 28 February 2011. (ARC is regulation strengthening initiative supported by the President’s Emergency Plan for AIDS Relief [27].) The nine-item survey contained questions about the stakeholder’s roles, responsibilities, and involvement with nursing and midwifery regulation. Survey questions also asked about the practice of task shifting (the term task sharing was not widely used yet) from physicians to nurses and midwives and queried respondents on what they perceived as the most important challenges to reforming nursing and midwifery regulation. Responses to survey questions were provided in the form of written answers, multiple-choice options, and rating agreement with statements on a Likert-type scale. The survey was anonymous; however, one question asked participants to voluntarily identify the country and the stakeholder group they represented.

Prior to taking the survey, all participants received a written description of the study and assurance regarding human subjects’ protection, reporting of results, and their rights to decline participation. A verbal overview of the survey was given, with time for questions and discussion. Written consent was waived in order to limit potential identification of survey participants. Completion of the survey served as non-verbal assent to participate in the study. Use of the survey was approved by the Institutional Review Board at the University of Washington and the CDC’s Associate Director for Science Office in the Division of Global HIV/AIDS.

## Results

A total of 32 stakeholders from 13 countries completed some or all of the survey, with approximately equal representation from each of the three stakeholder groups: 11 CNOs, 11 professional association presidents, and 10 academicians. Responses were tallied for each question separately and analyzed for comparison across stakeholder groups.

### Roles and activities

**Figure 1:**
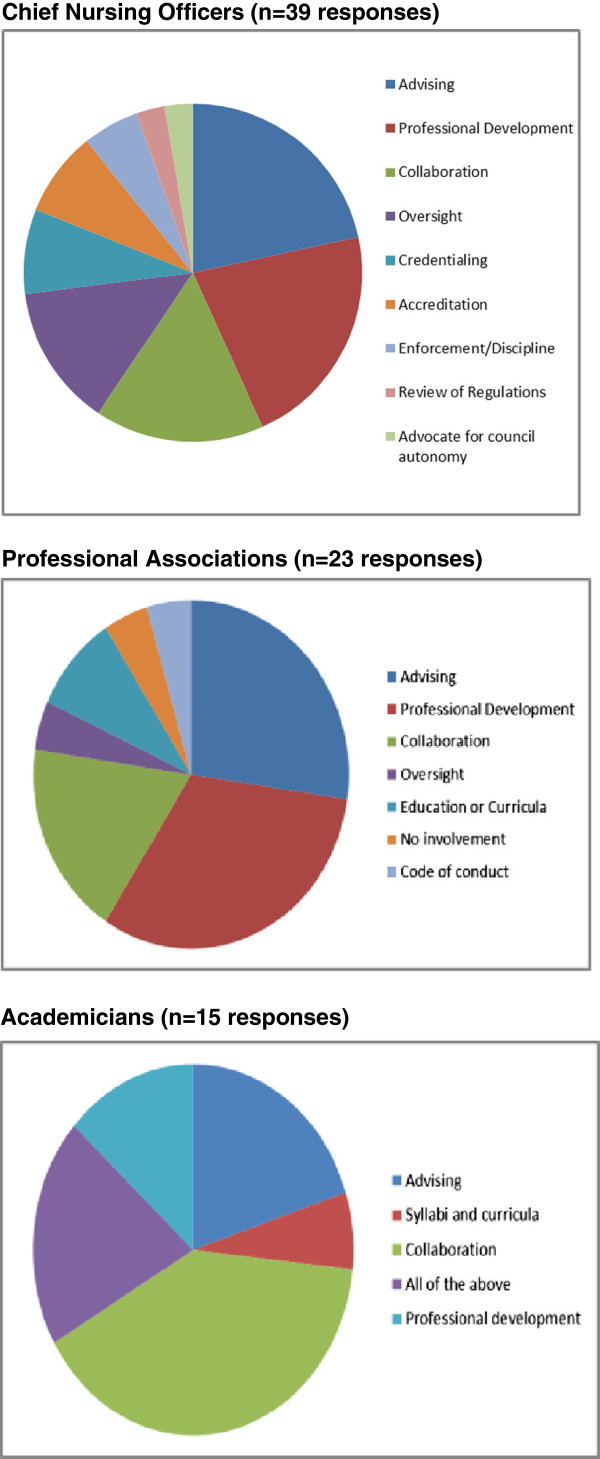
Stakeholder activities with the regulatory council, by group.

Stakeholders were asked to describe the role of their respective organization in nursing and midwifery regulation and to list the activities they engage in with the national regulatory council. There was a clear differentiation of roles according to the stakeholder group without much overlap of roles across the three groups (Table 1). In contrast to distinct roles, the regulation-related activities reported by the three groups showed substantial overlap (Figure 1). For example, all stakeholder groups listed ‘advising the council’ and ‘collaborating with other stakeholders’ with the highest frequency. Additionally, all three groups reported activities pertaining to the ‘professional development of nurses and midwives’.

**Table 1:** Roles of stakeholder groups in professional nursing and midwifery regulation

	**Chief nursing officers**	**Association presidents**	**Academicians**
**Role of stakeholder group in regulation**	9 respondents	10 respondents	8 respondents
	(82% response rate)	(91% response rate)	(80% response rate)
	• Advise on policy; supervise (5)	• Advocate on behalf of nurses (4)	• Advise on training standards and curricula (6)
	• Support the council (2)	• Collaborate and liaise between council and nurses (4)	• Facilitate collaboration between Ministry of Health and education institutions (2)
	• Ensure regulations protect the public (2)	• Ensure compliance with licensure and professional development (2)	

### Representation on and involvement with the council

Stakeholders were asked if their organization was represented on the council and how involved they felt in council decision-making (Table 2). The CNO group reported the highest representation on national nursing councils - 100% of CNO respondents indicated they were represented. Only 78% of academicians stated they had representation on the national council; 82% of association presidents said their education institution was represented. Respondents were also asked to rate their agreement with a statement that their organization’s input mattered and they were engaged by the council in decisions around nursing and midwifery regulation. The CNO group had the highest perception (90%) that their input mattered and that their ministry was engaged in council decisions; academicians were close behind at 87.5%. The association presidents had the lowest rating of agreement with the statement that their input mattered - only 60% agreed or strongly agreed while 40% were either neutral or disagreed. There was near complete accord within and across the different stakeholder groups that the primary means of communicating with the council was through their organizations’ activities with and representation on the council.

**Table 2:** Stakeholder representation on, input to, and communication with the nursing regulatory council

	**Chief nursing officers**	**Association presidents**	**Academicians**
**Representation of organization on the council**	Yes: 9 (100%)	Yes: 9 (82%)	Yes: 7 (78%)
	No: 0	No: 2	No: 2
**Agreement that organization’s input matters and are engaged in council decisions**	Strongly agree or agree: *n* = 9 (90%)	Strongly agree or agree: *n* = 6 (60%)	Strongly Agree or Agree: *n* = 7 (87.5%)
	Neutral: *n* = 1	Neutral or Disagree: *n* = 4	Neutral: *n* = 1
**Organization’s primary means of communication with the council**	Meetings and representation on the council: *n* = 8	Meetings and representation on the council: *n* = 11	Meetings and representation on the council: *n* = 8
	Direct communications: *n* = 1; Telephone: *n* = 1		Formal approvals of curricula: *n* = 1

### Task shifting and adapting regulations

**Figure 2:**
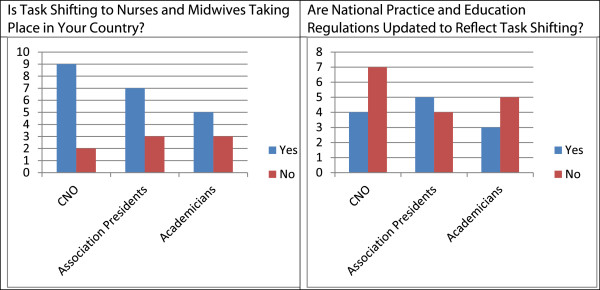
Perception of task shifting and currency of regulations, by stakeholder group.

Stakeholders were asked if nurses and midwives in their country performed task-shifted HIV-related services and if regulations (such as scope of practice) reflected this shift in service provision (Figure 2). The majority of all three groups stated that nurses and midwives were engaged in task shifting (the term used on the survey) for HIV-related services; however, only one-third of CNOs and association presidents felt the regulations in their country had been updated to reflect task shifting. A follow-up question asked stakeholders to list what their role was in updating regulations to reflect task shifting and educational reforms (Table 3). The responses were distinct for each stakeholder group. For example, the majority of CNOs stated their role in adapting regulations was to ‘support the council’; the association presidents most often stated their role in adapting regulations was to ‘collaborate in decision making’ and ‘keep the council informed’; and the academicians felt their primary roles were to ‘communicate with the council’ and ‘collaborate with stakeholders’.

**Table 3:** Stakeholder roles in adapting nursing and midwifery regulations

	**Organizational roles in adapting regulations**
**Chief nursing officers**	• Support the council in its functions
	• Give recommendations to the council
	• Review regulatory frameworks, education curricula, national policies, and strategies
**Association presidents**	• Collaborate with the council in decision making
	• Keep the council informed and updated regarding professional practice
	• Advocate for reform to education system
**Academicians**	• Communicate and collaborate with council and other stakeholders
	• Assist with upgrading lower nursing and midwifery cadres
	• Assess and score new nursing and midwifery colleges

### Challenges in regulations

**Figure 3:**
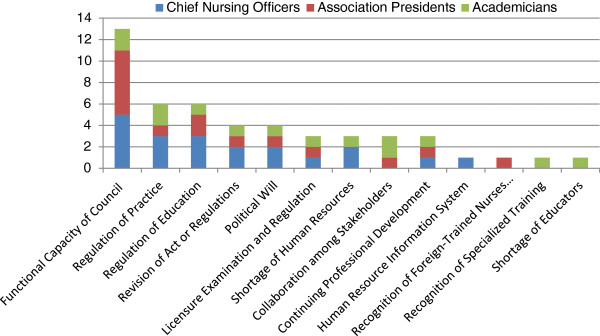
Most important issues or challenges facing nursing and midwifery regulation, by stakeholder group.

Stakeholders were asked to list the two most important issues or challenges in nursing regulation (Figure 3). The most important issue or challenge cited from all three groups was the ‘capacity of the council’ to carry out its regulatory functions. Of the 13 responses identifying the capacity of the council as the biggest challenge in national nursing and midwifery regulation, five identified the lack of autonomy of the council as the main challenge; three listed insufficient human resources on the council; another three cited insufficient expertise in council members; one response cited insufficient financial resources at the council; and one stated lack of recognition of the registrar’s role. The second most frequently-cited challenge related to the regulation of practice (for example, regulating private practice and monitoring professional conduct) and the regulation of education (for example, duration of pre-service preparation and ability to track students).

## Discussion

Task shifting and educating workers to safely and effectively assume additional responsibilities are uniformly recognized as vital components in realizing PEPFAR and other global health targets. However, our understanding of how best to strategically involve stakeholders in updating the nursing and midwifery regulation to support task shifting in sub-Saharan Africa is weak. We report the results of a survey of stakeholders in nursing and midwifery regulation from 13 countries in the ECSA region designed to increase our understanding of their roles and activities with regard to regulation. More than two-thirds of respondents stated that task shifting to nurses and midwives took place in their country, yet less than half stated that regulations for practice and education accounted for task shifting. These data suggest there may be gaps between the services provided by nurses and midwives and their authorized scopes of practice and the training that initially prepared them for practice.

The highest ranked challenge to transforming nursing and midwifery regulation in the ECSA region was the limited capacity of the national councils to carry out their regulatory functions. With task shifting and pre-service reform creating an urgency to update regulations, the perception that the capacity of the regulatory body is sub-optimal is concerning. If councils do not have the resources or capacity to create supportive regulatory frameworks for nursing practice, it could slow efforts to ensure the sustainability of task shifting and pre-service reform. Our data indicate that stakeholder groups play complementary roles in regulation with some redundancy in activities, suggesting that appropriately involved stakeholders could assist the council with implementing complicated or time consuming regulation activities, such as continuing professional development. However, our findings indicate that only 60% of association presidents felt engaged in regulation activities with the council, and only 78% of academicians felt adequately represented on the regulatory council. These findings suggest that councils could benefit from ensuring these stakeholder groups are represented on the council and more fully engaged in decision making related to task shifting and pre-service reform.

This study supports literature from the normative international nursing and midwifery groups with regard to the roles of stakeholder groups and their specific roles in creating or adapting regulations. The findings of this study are consistent with those from similar studies that investigated the challenges faced by CNOs in their role [28,29]. Our findings also confirm previous reports in the literature that the regulatory frameworks in some countries need updating. While guidelines on task shifting and recommendations on transforming health professional education exist, this study provides new evidence that countries in the ECSA region face obstacles to adapting their practice and education regulations accordingly. This study provides information for effectively engaging these leaders in regulatory reform by clarifying their roles, responsibilities, and activities regarding regulation overall as well as their specific perspectives on task shifting and pre-service reform. This evidence has reinforced the ARC initiative's approach of equally engaging these three stakeholders, along with the council, in support of concerted national regulatory reform efforts.

Limitations to this study include the small number of respondents (10 or 11 in each stakeholder group), potential selection bias because of the use of a convenience rather than representative sampling approach, and instances in which some respondents did not answer every question on the survey. As a result, the generalizability of findings is potentially limited by the personal or professional bias that may have influenced survey responses. Additionally, because of the open-ended nature of certain survey questions, some responses were unique to one individual, contributing to small modes and long tails in frequency of responses. This study was not able to clarify or solicit more information from survey respondents nor cross-check statements about task shifting and the currency of national regulations. While number of respondents from each group was not large, it nevertheless provides a regional representation of nurse and midwifery leadership opinion. It is also noteworthy that the responses were fairly consistent within groups, suggesting that the number of respondents was sufficient to capture a consensus within each respective stakeholder group.

The findings of this study have implications for the growing number of global health initiatives encouraging task shifting and global efforts to transform health professional education. Because wide-scale adoption, institutionalization, and sustainability of these workforce strategies depends on updating regulatory frameworks, it is essential that regulatory councils engage these key stakeholder groups at an early stage and recognize and clarify their roles and responsibilities on these issues. Amid calls to strengthen regulatory frameworks and engage regulatory councils, global health agencies may find that regulatory councils lack the necessary resources or capacity, as documented in our survey findings. Planning for the success of global health initiatives, including PEPFAR, will require capacity building of councils as well as greater involvement of stakeholders from the ministry of health, professional associations, and the education sector who can complement, advocate, and lend technical support to the diverse work of the council. Future research is needed to understand what regulatory changes task shifting and pre-service reform require and the barriers regulators face in attempting to make them. Additional studies could be enhanced by engaging these stakeholders to examine potential strategies to overcome barriers to reform and to measure the effectiveness of efforts aimed at building regulatory capacity.

## Conclusion

As the need for expanded nursing and midwifery functions grows, ensuring corresponding regulations are in place to govern professional practice is an increasing concern. This study suggests there is not alignment in some countries between the roles and responsibilities of practicing nurses and midwives and the current practice and education regulations. The need to advance regulations and education to enhance the sustainability of task shifting and pre-service reform was recognized by study participants. Barriers to regulatory and educational progress are reported to include insufficient capacity at the level of the nursing and midwifery council, which may lack the information technology or technical assistance required to rapidly adjust regulations to changing practice realities. The discrepancy between regulation, practice, and education identified by key leaders, coupled with their interest in bringing these into closer alignment, provides an important basis for further work in this area.

These findings also suggest that regulatory and educational advancement efforts benefit from the engagement of global initiatives that support expanded roles of nurses and midwives in the practice setting. Thus, it befits global partners, such as PEPFAR and WHO, to recognize that sustaining ongoing progress requires appropriate systems support to ensure both effective and consistent provision of services over time. Strengthening the capacity of the regulatory councils to carry out regulatory reform is an essential first step in advancing professional practice and transforming pre-service education. The findings of this study also suggest the value of engaging three key stakeholders groups, not only within countries, but on a regional basis, to share best practices, expertise, experiences, and other related resources.

## Endnotes

^a^ For the purposes of this paper, stakeholder is a term that encompasses those who are engaged in regulation in substantial ways either due to the responsibilities of their positions (legal or institutional) or by their roles in representing affected parties.

## Abbreviations

ARC: African health professions regulatory collaborative; ART: Antiretroviral therapy; CDC U.S: Centers for disease control and prevention; CNO: Chief nursing officer; ECSA: East, central, and southern Africa; HIV/AIDS: Human immunodeficiency virus/acquired immunodeficiency syndrome; MOH: Ministry of health; PEPFAR: President’s emergency plan for AIDS relief; WHO: World Health Organization

## Competing interests

The authors declare that they have no competing interests.

## Authors’ contributions

CM designed the study with substantial contributions from JV and PR. MS, JG, and MK made critical revisions to the manuscript for intellectual content; PR gave final approval of the version to be published. All authors read and approved the final manuscript.
